# Metabolomics-Based Machine Learning Models Accurately Predict Breast Cancer Estrogen Receptor Status

**DOI:** 10.3390/ijms252313029

**Published:** 2024-12-04

**Authors:** Kamala K. Arumalla, Jean-François Haince, Rashid A. Bux, Guoyu Huang, Paramjit S. Tappia, Bram Ramjiawan, W. Randolph Ford, Maria Vaida

**Affiliations:** 1 Department of Analytics, Harrisburg University of Science and Technology, Harrisburg, PA 17101, USA; karumalla@alumni.harrisburgu.edu (K.K.A.); marriotts2010@gmail.com (W.R.F.); 2 BioMark Diagnostic Solutions Inc., Quebec, QC G1K 3G5, Canada; jhaince@biomarkdiagnostics.com (J.-F.H.); ghuang@biomarkdiagnostics.com (G.H.); 3 BioMark Diagnostics Inc., Richmond, BC V6X 2W2, Canada; rahmed@biomarkdiagnostics.com; 4 Asper Clinical Research Institute, Winnipeg, MB R2H2A6, Canada; ptappia@sbrc.ca (P.S.T.); bramjiawan@sbrc.ca (B.R.); 5 Department of Pharmacology & Therapeutics, Max Rady College of Medicine, University of Manitoba, Winnipeg, MB R3E 0T6, Canada

**Keywords:** estrogen receptors, breast cancer, metabolomics, machine learning models

## Abstract

Breast cancer is a global concern as a leading cause of death for women. Early and precise diagnosis can be vital in handling the disease efficiently. Breast cancer subtyping based on estrogen receptor (ER) status is crucial for determining prognosis and treatment. This study uses metabolomics data from plasma samples to detect metabolite biomarkers that could distinguish ER-positive from ER-negative breast cancers in a non-invasive manner. The dataset includes demographic information, ER status, and metabolite levels from 188 breast cancer patients and 73 healthy controls. Recursive Feature Elimination (RFE) with a Random Forest (RF) classifier identified an optimal subset of 30 features—29 biomarkers and age—that achieved the highest area under the curve (AUC). To address the class imbalance, Gaussian noise-based augmentation and Adaptive Synthetic Oversampling (ADASYN) were applied, ensuring balanced representation during training. Four machine learning (ML) algorithms—Random Forest, Support Vector Classifier (SVC), XGBoost, and Logistic Regression (LR)—were evaluated using grid search. The Random Forest classifier emerged as the top performer, achieving an AUC of 0.95 and an accuracy of 93%. These results suggest that ML has great promise for identifying specific metabolites linked to ER expression, paving the development of a novel analytical tool that can minimize current challenges in identifying ER status, and improve the precision of breast cancer subtyping.

## 1. Introduction

Every breast cancer subtype can be characterized by histological and molecular features, and their intrinsic heterogeneity leads to an array of clinical presentations and different responses to therapy. Histological subtypes include mainly ductal and lobular carcinomas. The molecular classification of breast cancer can be abridged based on the presence or absence of hormone receptors for estrogen (ER) and progesterone (PR), as well as the human epidermal growth factor receptor 2 (HER2). Combining these classifications creates four main breast cancer subtypes. Luminal A tumors are ER-positive and PR-positive, but negative for HER2, while ER positive, PR negative, and HER2 positive are classified as Luminal B, which can be more aggressive than Luminal A. HER2-positive subtype is overexpressing the HER2 gene and lacking both hormone receptors (ER-negative and PR-negative). Finally, triple-negative breast cancer (TNBC) lacks all three established receptors (ER-negative, PR-negative, and HER2-negative).

Understanding the specific subtype based on hormone receptor status and HER2 is crucial mainly because estrogen, in its primary form of 17β-estradiol (E2), has a vital role in breast cancer development, particularly in postmenopausal women [1]. When estrogen binds to receptors located on the surface of the cancer cells, it triggers signals within the cell that promote growth and division. In general, cancer cells that express hormone receptors for estrogen (ER-positive) have a better prognosis than those that do not (ER-negative) [2]. About 80% of breast cancers in women and 90% of breast cancers in men are ER-positive, according to the National Cancer Institute. Patients with breast tumors that show a very low level of estrogen positivity have ER negativity (1–9%) and have different characteristics and outcomes compared to those with higher ER positivity. It appears that these patients may not benefit from hormone therapy [3,4].

Tumors are composed of diverse cell populations with varying characteristics. This diversity, known as tumor heterogeneity, allows tumors to evolve, adapt to treatment, and spread to other body parts. Different cancer cells within a tumor can cooperate to promote tumor growth and progression. Estrogen hormones, when interacting with estrogen receptors (ERs), can influence the development and progression of breast cancer. Disruptions in ER signaling can lead to uncontrolled cell growth [5]. ER+ breast cancers exhibit a metabolic profile characterized by oxidative phosphorylation, one-carbon metabolism, lipid synthesis, and amino acid metabolism. Estrogen signaling enhances mitochondrial efficiency and redox balance, promoting cell survival. In contrast, ER-negative breast cancers rely more on glycolysis, lipid oxidation, and branched-chain amino acid metabolism to meet their energy demands and combat oxidative stress. These distinct metabolic profiles have implications for targeted therapies. Targeting glycolysis or BCAA metabolism may be effective for ER− cancers, while ER+ cancers might be more sensitive to treatments targeting estrogen-driven lipid metabolism or oxidative phosphorylation [6].

Although cancer cells are dependent on estrogen for growth, women with ER-positive cancers have better outcomes if they lack a progesterone receptor (PR) and human epidermal growth factor receptor 2 (HER2) receptors (ER+/PR−/HER2−). ER-negative cancers include triple-negative breast cancer, or TNBC (ER−/PR−/HER2−), which has the poorest survival rate [7]. In addition, estrogen receptor (ER) status affects mortality risk differently for White and Black women with breast cancer [8]. White women with ER-positive tumors tend to have a higher risk of death, underscoring the complexity of hormone receptors in breast cancer [9]. Indeed, both hormone receptor variation as well as tumor heterogeneity may influence breast cancer treatment [10]. Discordance in the level of expression of hormone receptors can be a valuable indicator in developing non-invasive disease detection and targeted treatment methods [11]. The analysis of different metabolites in the body fluids can facilitate the subtyping of breast cancer and present a viable clinical application. In this regard, studies have found that metabolite levels vary between healthy people and breast cancer patients, thus providing promising scope for the early detection of this type of cancer, particularily in premenopausal women [12,13].

Traditional methods for classifying breast cancer subtypes have predominantly relied on invasive biopsies [14]. In clinical practice, estrogen receptor status in breast cancer is primarily determined using immunohistochemistry (IHC) and reverse transcription-quantitative polymerase chain reaction (RT-qPCR). IHC involves staining tumor tissues with antibodies specific to the estrogen receptor. RT-qPCR quantifies the mRNA expression levels of the ESR1 gene, providing a more objective and quantitative assessment of IHC. An ICH/RT-qPCR comparison study found that the AUC value for IHC was 0.95, while RT-qPCR achieved an AUC of 0.94 [15]. However, RT-qPCR requires high-quality RNA samples and specialized equipment, whereas IHC relies on biopsies which may limit its accessibility in some clinical settings. Given these limitations, integrating them with advanced analytical approaches, such as metabolomics, could enhance the accuracy and reliability of ER status determination.

While several studies have already been conducted to explore the possibility of using metabolite panels as surrogate biomarkers for early detection of breast cancer, many fall short looking at differential metabolic signature representatives of the distinct breast cancer subtypes. Machine learning tools in the past decade have been used for extensive breast cancer classification using traditional biopsy data [16]. As breast cancer subtype analyses are normally performed from biopsy material and subsequent histopathological analysis, a need for exploring non-invasive detection methods can save time and minimize costs associated with successive biopsy procedures. Recent advancements allow for the analysis of plasma, serum, saliva, and urine metabolites, which can offer new avenues for understanding the relationship between hormone receptor status and metabolite biomarkers in breast cancer subtyping [17]. The metabolomics platform has several distinct advantages including reliability and accuracy of the data, cost-effectiveness, and the rapid nature of the test. Metabolomics has the potential to detect metabolic imbalances, even at early disease onset, which enhances treatment outcomes. Identifying key small molecules can be crucial for differentiating between cancerous and healthy tissue and improving diagnosis accuracy [12].

Deep learning techniques, such as neural networks, and unsupervised machine learning methods are effective tools for analyzing complex biological data, such as patient metabolite profiles, to improve the precision of breast cancer diagnostics and subtyping. Neural networks have demonstrated superior performance in predicting breast cancer estrogen receptor (ER) status, achieving an area under the curve (AUC) of 0.93 [18]. Additionally, unsupervised methods, including Principal Component Analysis (PCA), K-means, Sparse K-means, Spectral Clustering, and SIMLR successfully identified biologically meaningful breast cancer subgroups. These models analyzed 499 metabolites from breast cancer patients with localized tumors, and distinguished between patients based on characteristics such as estrogen receptor (ER) status, molecular subtype, and metabolic activity [19].

Metabolomics, as an analytical method, is particularly suited for identifying molecular discrepancies among different cancer types due to its ability to capture a wide array of metabolic signatures. This approach, combined with non-invasive sampling techniques like serum analysis, suggests a viable strategy for routine clinical biomarker assessment, enhancing our understanding of cancer’s metabolic footprint [20]. Advanced statistical methodologies have the potential to unveil subtle metabolic patterns, which are essential given the complexity and noise in biological data. This complexity arises from factors such as cancer progression rates, genetic diversity among individuals, and the inherent overlap in metabolite signals [21]. Moreover, research involving machine learning analysis of blood serum metabolites has demonstrated its capability to differentiate between breast cancer patients and those without cancer, pointing towards a non-invasive diagnostic paradigm for breast cancer [22]. Given these advancements, our study aims to leverage metabolomics to pinpoint specific metabolic biomarkers associated with ER hormone receptor status in breast cancer patients.

## 2. Results

To assess differences in demographic and clinical variables between estrogen receptor (ER) status groups, statistical analyses were performed. Statistical distribution of demographic variables for estrogen receptor status is shown in Table 1. The mean age is higher for the ER-positive group (56.86 years old) compared to the ER-negative group (52.97 years old). The mean BMI is slightly lower for the ER-positive group than for the ER-negative group. The proportion of people with a history of smoking is lower for the ER-positive group than for the ER-negative group. The proportion of people who are current smokers is lower for the ER-positive group than for the ER-negative group. The proportion of people who are White is lower for the ER-positive group than for the ER-negative group. The proportion of people who are Black is similar for both groups. A two-sample t-test produced a p-value of less than 0.05 for the demographic variables of age and smoking history, indicating statistical significance. Similarly, two-sample t-tests for ER status, comparing groups with normally distributed variables, revealed p-values below the 0.05 significance threshold for 51 metabolites.There is a statistically significant effect size ranging from 2.0 to 7.31, indicating the difference between the means of the variables and ER expression of breast cancer data. One-way ANOVA results showed a statistically significant *p*-value of less than 0.05 and a large effect size difference range of 11.135–54.63 between the means of age and metabolite variables.

This study employed a comprehensive machine learning pipeline to optimize the classification performance of ER status through feature selection, data augmentation, and cross-validation techniques. Following data preprocessing, which included one-hot encoding and the removal of columns with a high percentage of missing values, the dataset was refined to 140 features. Among these, 8 were demographic variables, while the remaining 132 represented biomarkers. Recursive Feature Elimination (RFE) was employed with a Random Forest classifier to identify the most predictive features, reducing dimensionality and mitigating the risk of overfitting. An iterative approach evaluated feature subsets ranging from 10 to 40 to determine the optimal number of features. The best-performing feature set consisted of 30 features, including age and 29 biomarkers. To address the class imbalance, the pipeline employed two strategies: noise-based augmentation and Adaptive Synthetic Oversampling (ADASYN). Four machine learning algorithms, namely Random Forests, Support Vectors Classifier, XGBoost, and Logistic Regression, were assessed using hyperparameter tuning via grid search. For the RF model, the parameters tuned included the number of decision trees (n_estimators), set to [100, 200, and 500], and the maximum depth of the trees (max_depth), set to [none, 2, 5, 8, and 0]. For the SVC model, the grid search included the regularization strength (C), with values [0.1, 0.5, 1, 5], and kernel functions (kernel), with options for [linear, rbf, poly, sigmoid]. The XGBoost model was optimized over a broader grid, including the number of boosting rounds (n_estimators), set to [100, 200, 500], the maximum tree depth (max_depth), set to [2, 3, 5, 7, 10], and the learning rate (learning_rate), set to [0.01, 0.1, 0.001, 0.05]. Lastly, for LR, the grid search parameters included the regularization strength (C), with values [0.1, 0.5, 1], the penalty type (penalty), set to [l1, l2], and the optimization solver (solver), set to [lbfgs, liblinear].

Data augmentation and synthetic data generation techniques were performed in two phases. Initially, ER-positive class samples were augmented by adding Gaussian noise, creating slightly varied versions of existing data points. Specifically, Gaussian noise with a mean of 0 and a standard deviation of 0.01 was added to each feature. This generated 164 additional new data points that were similar to, but not identical to, the original samples, effectively increasing the diversity of the ER-positive class while preserving the underlying structure of the data. Additionally, Adaptive Synthetic Sampling (ADASYN) was applied during cross-validation to generate 164 new, synthetic samples for the minority class based on its distribution in the training feature space. Unlike traditional oversampling, ADASYN dynamically adjusts the generation process, focusing on samples that are harder to classify. These techniques ensured that the training dataset achieved a better balance between the majority and minority classes, reducing the risk of bias during model learning. After data augmentation, the dataset consisted of 600 records, with 328 ER-positive and 272 ER-negative samples.

The grid search optimization, combined with robust handling of class imbalance, was conducted within a stratified 5-fold cross-validation. The model performance was evaluated using the area under the receiver operating characteristic curve (ROC-AUC) as the primary metric, alongside precision, recall, F1-score, and accuracy. By iterating this process over 500 iterations, the methodology ensured that the best-performing model, hyperparameter configurations, and model performance were robustly identified.

**Figure 1:**
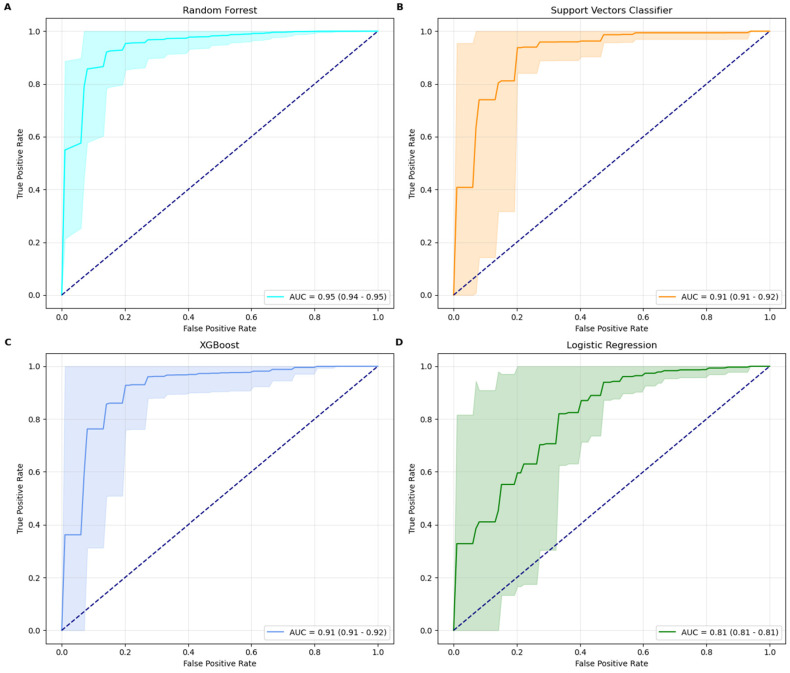
ROC-AUC curves for Random Forest (RF), Support Vectors Classifier (SVC), XGBoost, and Logistic Regression (LR). RF achieved the highest AUC of 0.95 (CI: 0.94-0.95 Panel (**A**)), followed by SVC (Panel (**B**)), XGBoost (Panel (**C**)), and LR (Panrl (**D**)).

Among the four machine learning models tested, the Random Forest classifier emerged as the best-performing model, achieving a ROC-AUC score of 0.95 and an accuracy of 93% with its optimal parameters: max_depth = none and n_estimators = 100. The model’s precision (94%) and recall (98%) demonstrate its robustness in minimizing false predictions while maintaining strong overall performance. RF performed in line with established IHC, while outperforming RT-qPCR methods [15]. The XGBoost model, configured with the best parameters learning_rate = 0.1, max_depth = 10, and n_estimators = 500, also demonstrated strong performance. It achieved an ROC-AUC score of 0.92, accuracy of 91%, precision of 93%, and recall of 96%. The SVC model, optimized with a rbf kernel and a regularization parameter C = 5, achieved a ROC-AUC score of 0.91, accuracy of 90%, precision of 91%, and recall of 97%. In comparison, the Logistic Regression model delivered the lowest metrics, with a ROC-AUC score of 0.81 and an accuracy of 81%. This performance was obtained using a regularization parameter (C) of 1, an l2 penalty, and the bfgs solver. While its precision (92%) is high, its recall (84%) suggests a higher tendency for false negatives, reducing its efficacy in identifying minority class instances. Random Forest classifier is therefore the most robust and effective model for this classification task, providing superior results across all metrics. XGBoost and SVC also performed well, demonstrating strong precision and recall, while LR, though more interpretable, is less competitive for this specific application due to its lower recall and ROC-AUC scores. AUC curves, along with their confidence intervals, are shown in Figure 1.

## 3. Discussion

**Figure 2:**
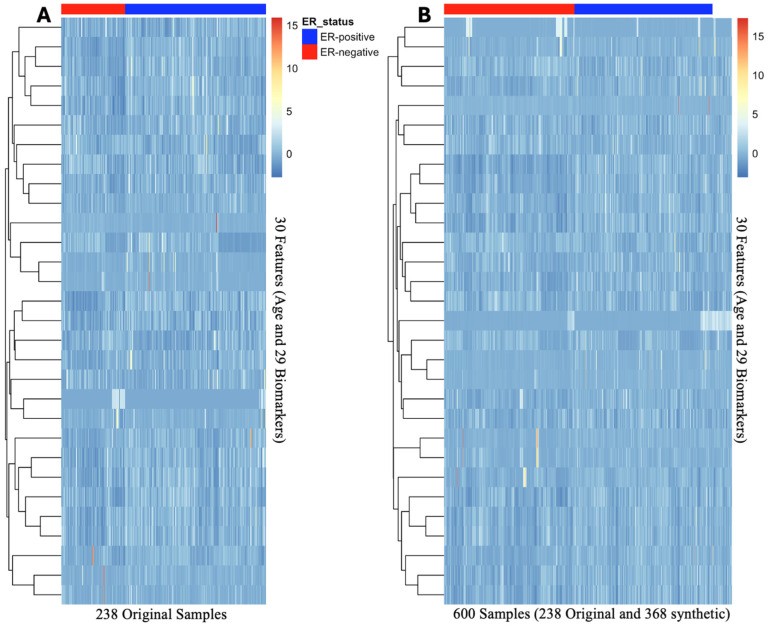
Heatmaps of 30 selected features before and after data augmentation. Panel (**A**) heatmap depicts the original 238 samples. Panel (**B**) illustrates augmented data (238 original samples and 362 synthetic data points). Panel (**B**) augmented heatmap retained the distribution and feature clustering displayed in Panel (**A**).

**Figure 3:**
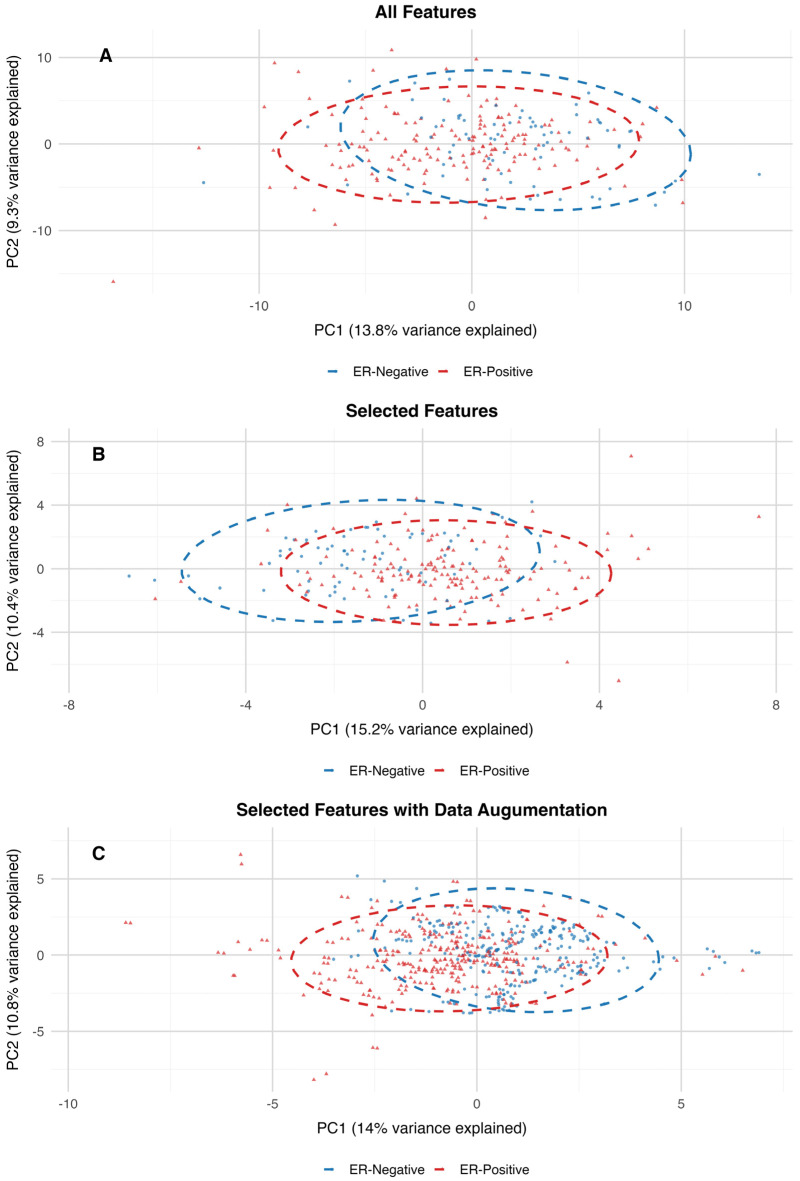
Principal Component Analysis plots of the original dataset with all features (Panel (**A**)), original dataset with 30 best-performing features (Panel (**B**)), and augmented dataset (Panel (**C**)). The dimensionality reduction patterns and variance explained match closely across all 3 projections.

This study demonstrated the effectiveness of using feature selection, data augmentation, and cross-validation to optimize classification performance. Recursive Feature Elimination with a Random Forest classifier successfully reduced the dataset from 140 features to 30 predictors, consisting of age and 29 biomarkers. To address the class imbalance, the study implemented a dual strategy: Gaussian noise augmentation and Adaptive Synthetic Sampling. The augmented data demonstrated similar feature clustering and heatmap distribution patterns when compared to the original dataset, as shown in Figure 2. Additionally, the PCA projection of the dataset with all features closely resembles the clustering patterns observed in the PCA projections of the 30-feature subset and the augmented dataset. The variance explained by the first two principal components is also consistent across these datasets, further supporting the alignment in their clustering structures (Figure 3).

To assess the differences between estrogen receptor (ER)-positive and ER-negative groups within high-dimensional metabolomics datasets, PERMANOVA (Permutation Multivariate Analysis of Variance) was utilized. The results of the PERMANOVA indicate that the centroids of groups differ significantly between ER-positive and ER-negative samples across all three datasets. For the dataset containing all features, the model explained 1.94% of the variance (R^2^ = 0.019) with an F-statistic of 4.68 and a *p*-value of 0.001. Although the variance explained is relatively small, the significant *p*-value indicates that the ER-positive and ER-negative groups have distinct distributions in the high-dimensional feature space. On the 30-features dataset, the model explained a slightly higher percentage of variance (R^2^ = 0.035) with an F-statistic of 8.51 and a *p*-value of 0.001. This increase in explained variance suggests that dimensionality reduction through feature selection enhanced the ability to distinguish between the two groups, likely by focusing on the most predictive variables. In the dataset with augmented features, the model explained a larger proportion of variance (R^2^ = 0.113) with an F-statistic of 75.64 and a *p*-value of 0.001. The increased R^2^ reflects the impact of data augmentation in amplifying the signal separating ER-positive and ER-negative groups, likely by addressing class imbalance and improving the model’s robustness. Therefore, by reducing the dimensionality of the features and introducing additional synthetic data, the variance explained increased, and the overall distribution of the data was preserved.

Breast cancer subtyping, determined by hormone receptor levels such as estrogen receptor (ER), progesterone receptor (PR), and human epidermal growth factor receptor 2 (HER2), is crucial for predicting patient survival rates [23]. Comparative studies of machine learning (ML) models applied to breast cancer data, including clinical and imaging information, have shown that models like Random Forest and Support Vector Machines often outperform Logistic Regression, aligning with our findings [24,25,26]. Different ML models may excel in specific applications. For instance, Support Vector Machines (SVM) have shown promise in diagnosis, while Artificial Neural Networks (ANNs) tend to be more effective in predicting prognosis [27]. In a study involving data from 431 breast cancer patients, RF outperformed K-nearest neighbors, Naive Bayes, SVM, LR, and Multilayer Perceptron in personalizing predictions for response to neoadjuvant chemotherapy (NAC) before surgery. The RF model achieved an area under the curve of 0.88, significantly higher than LR’s 0.64, highlighting ML’s potential to optimize NAC treatment decisions. By incorporating diverse variables such as menopause status, hormone receptor levels (ER, PR, HER2), tumor grade, size, lymph node involvement, and the presence of inflammatory breast cancer, the RF model more accurately predicted patients likely to achieve a complete pathological response to NAC. [28]. The RF model in this study outperformed a deep learning approach in predicting ER status, which achieved an AUC of 0.93 [18]. While both studies aimed to predict ER status using metabolomics data on similarly sized datasets, the deep learning model utilized tissue samples without feature selection or data augmentation.

Beyond age, other researchers have highlighted additional demographic factors linked to breast cancer. A study analyzing data from the California Cancer Registry focused on triple-negative breast cancer examined 271 breast cancer tissue samples, including 204 that were ER-positive and 67 that were ER-negative. Using a combination of statistical methods and machine learning techniques such as RF and SVM, the researchers discovered that women with TNBC were more likely to be non-Hispanic Black and to reside in low-socioeconomic areas. Furthermore, this group tended to be diagnosed with larger, poorly differentiated tumors at later stages [29]. Additionally, African American women diagnosed with ER-positive breast cancer face a 40% higher risk of death compared to non-Hispanic White women with the same diagnosis [30]. These findings highlight the complex interplay of biological, socioeconomic, and racial factors in breast cancer outcomes.

Estrogen receptor (ER+/ER−) status is essential for the molecular classification of breast cancer. Exploring the relationships between distinct metabolites from various pathways offers an exciting and non-invasive avenue for developing robust machine learning methods in this area. Previous research has sought to establish connections between metabolomics and breast cancer subtypes [31,32,33]. This study expands on existing approaches by developing efficient models that integrate hormone receptor data with metabolomic profiles. To this extent, we were able to identify a subset of 29 metabolites specific to the ER, which is crucial for breast cancer subtyping.

Current treatments for breast cancer, including hormone therapy, targeted therapies, and chemotherapy, often fail due to resistance. A key factor contributing to this resistance is metabolic reprogramming, where cancer cells change their metabolic processes to survive and grow [34]. Immunohistochemistry is the current standard for diagnosing ER status in breast cancer from tumor samples [35]. A metabolomics-based approach offers a potential solution to monitor ER status dynamically. Machine learning models can accurately classify tumors as ER-positive or ER-negative by analyzing a patient’s metabolic profile. This approach can be particularly useful in ambiguous cases or when traditional methods like IHC are inconclusive. Routine metabolomic profiling and machine learning analysis can enable real-time monitoring of ER status, allowing for timely adjustments to treatment plans.

In conclusion, our study focused on expanding the scope of the existing literature by exploring ML models for different ER status. The determination of ER status is an important step in the management of breast cancer, influencing treatment options, providing prognostic information, enabling personalized medicine, and helping in the classification of the cancer subtype. The approach employed in this study effectively combined synthetic data generation, feature selection, and a Random Forest machine learning method to analyze metabolomics data specific to the estrogen receptor status. These steps enhanced the model’s ability to handle class imbalance, reduce dimensionality, and improve predictive accuracy Identified metabolites play a crucial role in energy production, amino acid synthesis and degradation, lipid metabolism, and nucleotide synthesis. Further analysis and larger cohorts are warranted to validate these metabolites as potential therapeutic strategies for different breast cancer subtypes. Hormone receptors like ER, PR, and HER2 also play crucial roles in ovarian cancers; hence, metabolomics profiling can extend the arena to screen diverse cancers. This approach highlights the importance of using data from multiple sources, including demographics and metabolomics, to better understand health status and potentially improve the clinical management of breast cancer.

A metabolomics-based approach for determining ER status has the potential to be more cost-effective. By analyzing non-invasive samples like serum or urine, it can reduce the need for invasive biopsies. Advancements in analytical techniques, such as mass spectrometry and NMR, could enable faster and more efficient testing, especially in large-scale settings. Frequent monitoring of ER status using non-invasive metabolomics could potentially decrease the need for repeated biopsies over time. A comprehensive metabolomics platform can provide valuable information on tumor staging, subtyping, and receptor status in a single test, making it a promising approach for improving patient care and reducing healthcare costs.

## 4. Materials and Methods

### 4.1. Participants and LC-HRMS Analysis

The dataset used for this study included a total of 261 plasma samples from 188 patients with biopsy-confirmed breast cancer and 73 plasma samples from healthy volunteers. All biospecimens were obtained from the Cooperative Human Tissue Network (CHTN) biobank and were approved by the Institutional Review Boards. From a histological standpoint, the cancer cases consisted of 41 lobular carcinoma samples and 144 ductal carcinoma samples. Nearly 90% of the cancer patients were in stages I (98 patients) and II (70 patients), while the remaining 17 patients were classified as stage III. CHTN indicates that the tubes are kept at room temperature once collected and before processing and were processed within 2 to 4 h of collecting and freezing the aliquoted samples on dry ice or freezing them in vapor phase liquid nitrogen. The archived plasma samples from CHTN frozen (−80 °C) aliquots of 200–400 μL of plasma were assembled and shipped to The Metabolomics Innovation Centre (TMIC) at the University of Alberta, Canada, for quantitative metabolomic analysis. The cancer samples had detailed data on cancer stage, breast cancer histology, diagnosis, receptor status, chart reviews, age, body mass index, smoking status (never/former/current), race, and medical condition history. Healthy volunteers of comparable age with no significant health conditions were selected.

Demographic information for all the participants and clinical diagnosis of study subjects are summarized in Table 1. The specific information for breast cancer patients included the histology group with the location and type of the tumor. Categories included ductal, invasive mammary carcinoma, lobular, and malignant carcinoid tumor. The demographic dataset included ER, PR, and HER2 receptor status information collected from breast cancer patients. A targeted quantitative analysis of 137 metabolites was performed by DI-LC/MS/MS assay on plasma samples from breast cancer patients and healthy participants.

### 4.2. Analytical Procedures

A targeted, quantitative mass spectrometry (MS)-based metabolomics approach was undertaken to analyze 138 metabolites in the plasma samples by DI-LC/MS/MS using the TMIC (The Metabolomics Innovation Centre, Edmonton, AB, Canada) PRIME assay as previously described. Mass spectrometric analysis of the diluted extracts was performed on an HPLC (Agilent 1260 HPLC, Agilent Technologies, Santa Clara, CA, USA) equipped with a Qtrap^®^ 4000 tandem mass spectrometry instrument (Applied Biosystems/MDS Analytical Technologies, Foster City, CA, USA). This assay enables the targeted identification and quantification of up to 138 different endogenous metabolites, including amino acids, acylcarnitines, biogenic amines and derivatives, organic acids, uremic toxins, glycerophospholipids, sphingolipids, and sugars. The method employs chemical derivatization (via 3-NPH for organic acids or PITC for amine-containing compounds), analyte extraction and separation, and selective mass-spectrometric detection using multiple reaction monitoring (MRM) pairs for metabolite identification and quantification. Isotope-labeled ISTDs (internal standard spiking solution), along with other ISTDs, are used for accurate metabolite quantification.

### 4.3. Stock Solutions, Internal Standard (ISTD) Mixture, and Calibration Curve Standards for Metabolomic Assays

All solid chemicals were carefully weighed on a CPA225D semi-microelectronic balance (Sartorius, New York, NY, USA) with a precision of 0.0001 g. Stock solutions of each compound were prepared by dissolving the accurately weighed solids in double-distilled water. Calibration curve standards were obtained by mixing and diluting the corresponding stock solutions with double-distilled water. For amino acids, biogenic amines, carbohydrates, carnitines and derivatives, and phosphatidylcholines and their derivatives, stock solutions of isotope-labeled compounds were also prepared in the same way. A working internal standard (ISTD) solution mixture in water was also made by mixing all the prepared isotope-labeled stock solutions together. For organic acids, stock solutions of isotope-labeled compounds were prepared by dissolving the accurately weighed solids in 75% aqueous methanol. A working internal standard (ISTD) solution mixture in 75% aqueous methanol was made by mixing and diluting all the isotope-labeled stock solutions. All standard solutions were aliquoted and stored at −80 °C until further use.

### 4.4. Sample Preparation and Liquid Chromatography/Direct-Injection Mass Spectrometry for Metabolomic Assays

A targeted, quantitative mass spectrometry (MS)-based metabolomics approach was used to analyze the plasma samples using a combination of direct-injection (DI) MS and reverse-phase high-performance liquid chromatography (HPLC) tandem mass spectrometry (MS/MS). This 96-well plate, semi-automated assay, in combination with an ABI 4000 Q-Trap (Applied Biosystems/MDS Analytical Technologies, Foster City, CA, USA) mass spectrometer, can be used for the targeted identification and quantification of up to 138 different endogenous metabolites including amino acids, organic acids, biogenic amines, acylcarnitines, glycerophospholipids, sphingolipids, and sugars. The method combines the derivatization and extraction of the 138 analytes, and the selective mass-spectrometric detection using multiple reaction monitoring (MRM) pairs. Isotope-labeled internal standards and other internal standards are integrated into special filter inserts placed inside a 96-well plate for precise metabolite quantification. The assay uses an upper 96 deep-well plate with a 96-well filter plate attached below using sealing tape. The first 14 wells in the upper plate are used for quality control and calibration. The first well serves as a double blank, three wells contain blank samples, seven wells contain reference compound standards, and three wells contain quality control samples.

Briefly, plasma samples were thawed on ice (in the dark), vortexed, and centrifuged at 18,000 rcf (relative centrifugal force or × *g*). Then, 10 µL of each sample was loaded onto the center of the filter inserted on the upper 96-well kit plate, and dried in a stream of nitrogen. Subsequently, PITC was added to each well (in the plate) for amine derivatization. After incubation, the filter inserts were dried using an evaporator. Extraction of the metabolites was then achieved by adding 300 µL of methanol containing 5 mM ammonium acetate. The extracts were obtained by centrifugation (at 50 rcf for 5 min) of the double plate system. This allowed the contents of the upper 96-well plate to flow into the lower 96-deep well plate. For analysis of biogenic amines and amino acids, extracts were then diluted by water. For analysis of sugars, carnitines, and lipids, extracts were diluted with methanol. Mass spectrometric analysis of the diluted extracts was performed on an HPLC (Agilent 1100 HPLC, Agilent Technologies, Santa Clara, CA, USA) equipped Qtrap^®^ 4000 tandem mass spectrometry instrument (Applied Biosystems/MDS Analytical Technologies, Foster City, CA, USA).

For the analysis of organic acids, 50 μL of the plasma samples were mixed thoroughly with the ISTD mixture solution and ice-cold methanol and then left in a −20 °C freezer overnight for protein precipitation. After removing the samples from the freezer, all the tubes were centrifuged at 18,000 rpm for 20 min (to spin down the protein precipitate). The supernatant was then transferred to each well of the 96-well plate system, followed by the addition of 25 μL each of the following three reagents: 3-NPH (250 mM in methanol), EDC (150 mM in methanol), and pyridine for a 2 h derivatization reaction. After the derivatization reaction was complete, water and a BHT solution (2 mg/mL in methanol) were added to dilute and stabilize the final solution. Finally, 10 μL was injected into an HPLC-equipped Qtrap^®^ 4000 mass spectrometer for LC-MS/MS analysis.

### 4.5. Data Processing

During data preprocessing, male participants were excluded from the dataset, as they constituted less than 1% of the total breast cancer population (23 records), ensuring that the analysis focused on a homogenous group. Metabolites with more than 20% missing values were removed to maintain data quality, while missing values for other metabolites were replaced with their respective detection limit values to preserve their analytical relevance. Demographic variables with missing data were imputed using mean values, enabling a comprehensive dataset without compromising the integrity of the information. Categorical variables such as smoking history and race were cleaned and one-hot encoded to facilitate analysis. Smoking history was categorized into “never”, “former”, and “current”, with missing values replaced by “never” as a conservative assumption. Race was similarly encoded into “White”, “Black”, and “Other” categories. Continuous variables, including BMI and age, were standardized using z-scores for consistent scaling across the dataset.

## Figures and Tables

**Table 1:** Statistical distribution of demographic variables for estrogen receptor status. ER-positive, (has estrogen binding capacity); ER-negative (no estrogen binding capacity); current smokers are those who smoke on either a daily or an occasional basis; never smoker defined as an individual who has never smoked; former smokers (i.e., do not currently smoke) are individuals who had quit smoking within the past six months (inclusive) prior to study participation. SD, standard deviation.

Total Cases (*n* = 238)		ER-Positive	ER-Negative	*p*-Value
Population	Breast Cancer (*n* = 185)	164	21	
	Healthy (*n* = 53)	0	0	
Race	Black	11	7	>0.05
	White	147	64	
	Other	6	3	
Smoking	Current	18	5	<0.05
	Former	46	14	
	Never	99	54	
	Not Stated	1	1	
Age	Mean (SD)	56.86 (12.42)	52.97 (13.46)	<0.05
BMI	Mean (SD)	29.77 (7.28)	30.30 (7.51)	>0.05

## Data Availability

All data generated or analyzed during this study are included in this publication. Raw data are not publicly available due to privacy or ethical restrictions.
